# Integration of Single-Cell RNA Sequencing and Bulk RNA Sequencing Reveals That TAM2-Driven Genes Affect Immunotherapeutic Response and Prognosis in Pancreatic Cancer

**DOI:** 10.3390/ijms241612787

**Published:** 2023-08-14

**Authors:** Yan Du, Shi Dong, Wenkai Jiang, Mengyao Li, Wancheng Li, Xin Li, Wence Zhou

**Affiliations:** 1 The Second School of Clinical Medicine, Lanzhou University, Lanzhou 730030, China; duyan19951212@163.com (Y.D.); dongsh20@lzu.edu.cn (S.D.); jiangwk21@lzu.edu.cn (W.J.); limengyao_dsa@163.com (M.L.); 18993895902@163.com (W.L.); 2 Department of General Surgery, Lanzhou University Second Hospital, Lanzhou 730030, China

**Keywords:** pancreatic cancer, macrophages M2, immunophenotype, prognosis, immune checkpoint inhibitor

## Abstract

Tumor-associated macrophages M2 (TAM2), which are highly prevalent infiltrating immune cells in the stroma of pancreatic cancer (PC), have been found to induce an immunosuppressive tumor microenvironment, thus enhancing tumor initiation and progression. However, the immune therapy response and prognostic significance of regulatory genes associated with TAM2 in PC are currently unknown. Based on TCGA transcriptomic data and single-cell sequencing data from the GEO database, we identified TAM2-driven genes using the WGCNA algorithm. Molecular subtypes based on TAM2-driven genes were clustered using the ConsensusClusterPlus algorithm. The study constructed a prognostic model based on TAM2-driven genes through Lasso-COX regression analysis. A total of 178 samples obtained by accessing TCGA were accurately categorized into two molecular subtypes, including the high-TAM2 infiltration (HMI) cluster and the low-TAM2 infiltration (LMI) cluster. The HMI cluster exhibits a poor prognosis, a malignant tumor phenotype, immune-suppressive immune cell infiltration, resistance to immunotherapy, and a high number of genetic mutations, while the LMI cluster is the opposite. The prognostic model composed of six hub genes from TAM2-driven genes exhibits a high degree of accuracy in predicting the prognosis of patients with PC and serves as an independent risk factor. The induction of TAM2 was employed as a means of verifying these six gene expressions, revealing the significant up-regulation of BCAT1, BST2, and MERTK in TAM2 cells. In summary, the immunophenotype and prognostic model based on TAM2-driven genes offers a foundation for the clinical management of PC. The core TAM2-driven genes, including BCAT1, BST2, and MERTK, are involved in regulating tumor progression and TAM2 polarization, which are potential targets for PC therapy.

## 1. Introduction

Pancreatic cancer (PC) is a malignant disease with a high mortality rate. The prevalence of PC has increased by approximately 0.4% annually due to the growth of the global population and the increasing number of public health epidemics [1]. The 5-year overall survival (OS) rate for PC is observed to be less than 10%, positioning it as the seventh leading cause of cancer-associated mortality worldwide [2]. Although surgery presents the possibility of treatment, the number of cases that can be resected at the time of diagnosis is less than 20%, and the majority of patients who undergo surgery pass away as a result of local recurrence and/or metastasis [3,4]. Furthermore, despite the extensive utilization of innovative therapeutic approaches such as neoadjuvant therapy, gemcitabine/nab-paclitaxel, and FOLFRINOX chemotherapy, therapeutic efficacy remains limited, and the development of chemoresistance contributes to a poor prognosis [5,6]. Consequently, a pressing necessity exists to ascertain the efficacious prognostic factors for PC.

The tumor microenvironment (TME) in PC is defined by a dense stroma and extensive immunosuppression [7], which hinder drug delivery to malignant lesions by restricting blood flow. This leads to resistance to chemotherapy and the suppression of the antitumor immune response [8]. Tumor-associated macrophages (TAMs) are among the first infiltrating cells that exist within intraepithelial pancreatic malignancies, and their numbers increase throughout the progression to invasive cancer [9]. TAMs enhance cancer progression through diverse mechanisms such as immune suppression, angiogenesis, invasion, metastasis, and drug resistance [10]. The employment of the immune checkpoint blockade is now recognized as a viable therapeutic approach for individuals afflicted with advanced PC. Although immune checkpoint inhibitors (ICI) have shown encouraging therapeutic outcomes in multiple cancer types, their efficacy in the context of PC has proven to be less than satisfactory. TAMs are one of the significant contributors to PD-L1, thereby impeding the infiltration and efficacy of cytotoxic T cells. This process ultimately leads to unfavorable resistance to neoadjuvant immunotherapy [11]. The density of TAMs has been identified as an independent prognostic factor in patients suffering from PC and is correlated to an increased risk of disease progression, recurrence, metastasis, and shorter OS [12].

**Figure 1:**
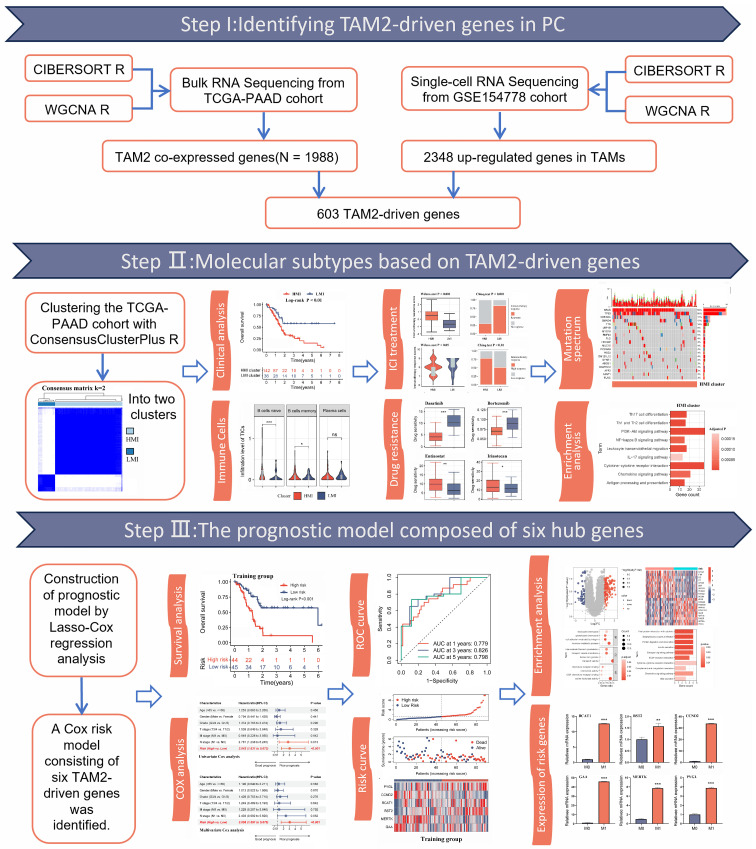
The workflow of our research. WGCNA, weighted correlation network analysis; TAM2, tumor-associated macrophages M2; TAMs, tumor-associated macrophages; ICI, immune checkpoint inhibitors. *, *p* < 0.05; **, *p* < 0.01; ***, *p* < 0.001; ns, no significance.

TAMs predominantly polarize towards a pro-tumoral M2 phenotype [13]. The infiltration characteristics of tumor-associated macrophages M2 (TAM2) directly affect the efficacy of ICI and the prognosis of PC patients. Therefore, clarifying the infiltration characteristics and molecular features of TAM2 can help provide more effective and personalized treatment strategies for PC patients. In this study, we identified TAM2-driven genes by analyzing the gene modules exhibiting a strong positive correlation with TAM2 infiltration in the TCGA-PAAD dataset and the highly expressed genes in TAMs based on the single-cell sequencing dataset. A prognostic model was constructed based on the identification of two distinct clusters, namely cluster high-TAM2 and cluster low-TAM2, using the TAM2-driven genes. Subsequently, a comparative analysis was performed on the prognosis, immune cell infiltration, immunotherapy response, and mutation profiles of different clusters, in addition to evaluating the predictive performance of the prognostic model. Subsequently, six TAM2-driven genes were validated as being closely associated with the M2 polarization of TAMs. These genes could potentially serve as a novel target in PC progression. The flowchart of this study is illustrated in Figure 1.

## 2. Results

### 2.1. Identification of TAM2 Co-Expressed Genes in PC

**Figure 2:**
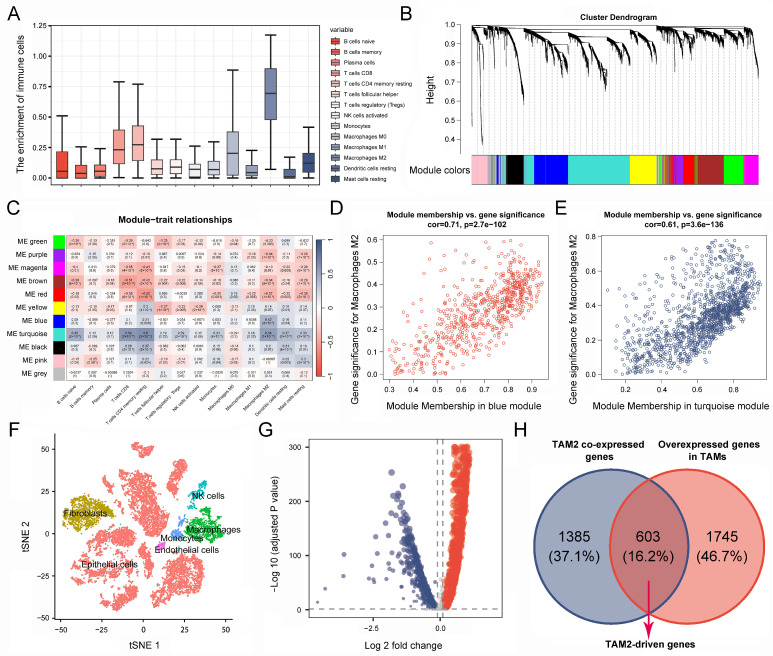
Identification of TAM2-driven genes. (**A**). Infiltration level of TICs in pancreatic cancer samples was calculated using the CIBERSORT algorithm. (**B**). Gene dendrogram and module colors based on WGCNA analysis, and different colors represent different clustering modules. (**C**). Correlation between gene module and TIC infiltration. (**D**,**E**). Correlation between gene significance and module membership. (**F**). Cluster analysis of single-cell sequencing samples. (**G**). The volcano plot illustrates the differentially expressed genes of tumor-associated macrophages, wherein the red color signifies upregulated genes, while the blue color signifies downregulated genes. (**H**). Genes common to both gene sets are defined as TAM2-driven genes in Venn diagram. TAM2, tumor-associated macrophages M2; TICs, tumor-infiltrating immune cells.

Based on the TCGA-PAAD cohort, we calculated the infiltration of tumor infiltrating immune cells (TICs) in 178 PC patients, revealing that TAM2 was significantly enriched in the TME of PC (Figure 2A). Given that TAM2 contributes to promoting tumor angiogenesis, immunosuppression, and hypoxia induction, we screened TAM2 co-expressed genes using WGCNA. The cluster dendrogram revealed the presence of 11 distinct modules. Significantly, the gray modules comprised genes that exhibited no aggregation with any other modules (Figure 2B). The relationship between the modules and TIC infiltration level was further analyzed. The results showed that the blue and turquoise modules had a significant positive relation to TAM2 (Figure 2C). Furthermore, we found that genes highly associated with TAM2 were key genes of the blue module or the turquoise module by analyzing the correlation between module membership and gene significance (Figure 2D,E). Finally, the 1988 genes included in the blue and turquoise modules were defined as TAM2 co-expressed genes (Supplementary Table S1).

### 2.2. Screening for TAM2-Driven Genes at the Single-Cell Level

RNA-seq data originated at the tissue level cannot clearly distinguish between tumor cells and stromal cells, while PC contains a high enrichment of stromal cells, and cancer cells represent only 5–10% of the tumor. Therefore, we further screened for co-expressed genes directly associated with TAM2 using single-cell sequencing data. All cells were classified into five categories and annotated as epithelial cells, fibroblasts, TAMs, NK cells, and monocyte endothelial cells, respectively (Figure 2F). Through comparative analysis of the gene expression between TAMs and other cell types, it was observed that a total of 2348 genes were significantly up-regulated in TAMs (Figure 2G, Supplementary Table S2). TAMs in PC mainly exhibit a TAM2 phenotype, so we took the intersection of TAM2 co-expressed genes and TAMs highly expressed genes. Finally, we obtained 603 TAM2-driven genes (Figure 2H, Supplementary Table S3).

### 2.3. Molecular Typing Based on TAM2-Driven Genes

**Figure 3:**
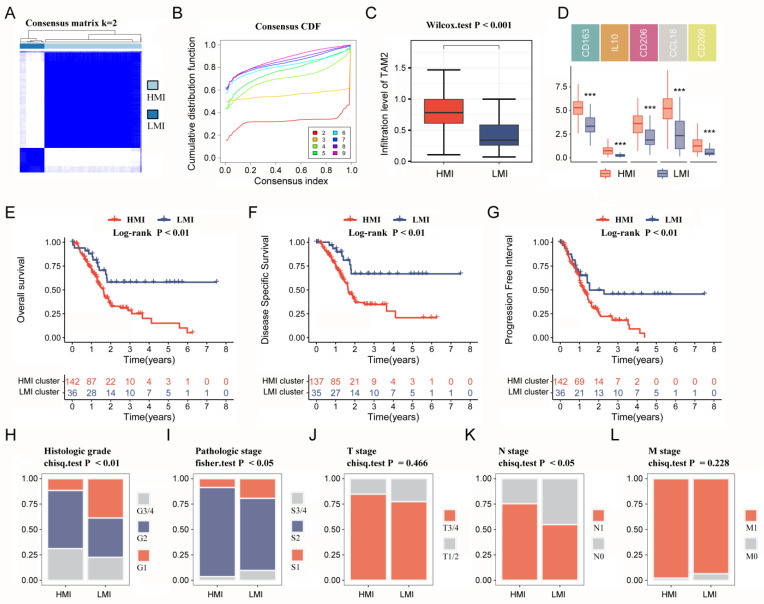
Immune clusters based on TAM2-driven genes. (**A**). Clustering heat map of PC samples when consensus k = 2. (**B**). The cumulative distribution frequency curve of PC samples. (**C**). Comparison of TAM2 infiltration levels in different clusters using the CIBERSORT algorithm. (**D**). Comparing the expression of TAM2 markers in different clusters. (**E**–**G**). OS time, DSS time and PFI time KM curves of PC patients with different clusters. (**H**–**L**). Percentage distribution of clinical characteristics in PC samples grouped by different clusters. TAM2, tumor-associated macrophages M2; PC, pancreatic cancer; CDF, cumulative distribution frequency; OS, overall survival; DSS, disease specific survival; PFI, progression-free interval; KM, Kaplan–Meier. ***, *p* < 0.001.

The univariate COX survival analysis results revealed that 127 genes in the TAM2-driven genes were strongly associated with PC prognosis, and were used for the next clustering analysis (Supplementary Table S4). Next, the 178 PC samples in the TCGA-PAAD cohort were clustered utilizing ConsensusClusterPlus, determining the optimal cluster numbers relying on the cumulative distribution function (CDF). The CDF area curve shows that the clustering result is relatively stable at k = 2, and all samples can be divided into two clusters (Figure 3A,B). These two clusters differed significantly in TAM2 infiltration (Figure 3C), so clusters 1 and 2 were named the HMI and LMI clusters, respectively. Furthermore, the expression of TAM2 cellular markers was assessed between the two clusters, demonstrating that five molecules exhibited notably higher expression in the HMI cluster (Figure 3D). In order to clarify the clinical value of clusters, we further evaluated the clinical characteristics and prognosis of different clusters. The analysis of survival, which was predicated on OS, disease-specific survival (DSS), and progression-free interval (PFI), showed a poor prognosis for the HMI cluster, while the LMI cluster had a favorable prognosis (Figure 3E–G). By comparing the clinical characteristics of different clusters, we found that PC patients with the HMI cluster had more pronounced tumor malignant manifestations, including a higher percentage of G3/4 in the histological grade and a higher percentage of N1 in the N stage (Figure 3H–L). Low tumor differentiation and lymph node invasion levels in patients with HMI clusters are among the reasons for their poor prognosis.

### 2.4. Immune Infiltration Analysis and Immunotherapy Prediction

**Figure 4:**
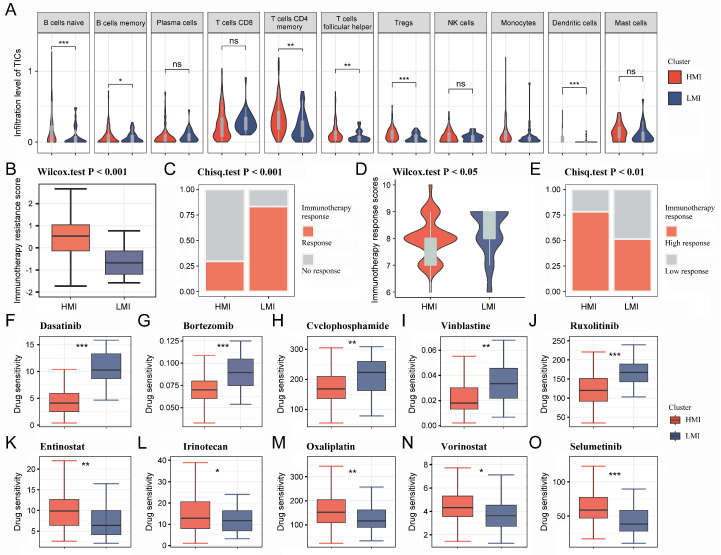
Immune infiltration analysis and immunotherapy prediction for HMI cluster and LMI cluster. (**A**). Violin diagram demonstrating TIC infiltration in different clusters using the CIBERSORT algorithm. (**B**,**C**). Immunotherapy resistance was predicted for each cluster utilizing the TIDE platform. (**D**,**E**). Immunotherapy response was predicted for each cluster utilizing the TCIA database. (**F**–**O**). IC50 values of 10 chemotherapeutic agents in PC patients with different clusters. HMI, high-TAM2 infiltration; LMI, low-TAM2 infiltration; TICs, tumor-infiltrating immune cells; TIDE, tumor immune dysfunction and exclusion; TCIA, the Cancer Immunome Atlas; IC50, half maximal inhibitory concentration. *, *p* < 0.05; **, *p* < 0.01; ***, *p* < 0.001; ns, no significance.

Considering the mutual cross-linking between TAM2 and other TICs in the TME, the TIC infiltration went through a further comparison in different clusters. Multiple TICs were significantly enriched in the HMI cluster, including naive B cells, memory B cells, memory CD4 T cells, follicular helper T cells, and dendritic cells (Figure 4A). TME is a key factor affecting the effectiveness of immunotherapy, so we further analyzed the immunotherapy response of different clusters. The results relying on the tumor immune dysfunction and exclusion (TIDE) database showed that the immunotherapy resistance score was higher in the HMI cluster (Figure 4B), and the immunotherapy response rate was lower in the HMI cluster (Figure 4C). According to the Cancer Immunome Atlas (TCIA) database, the HMI cluster had lower immunotherapy response scores than the LMI cluster (Figure 4D,E), suggesting that TAM2 may contribute to the mechanism of immunotherapy resistance in PC.

### 2.5. Targeted Drugs Sensitivity Analysis and Mutation Profiling

**Figure 5:**
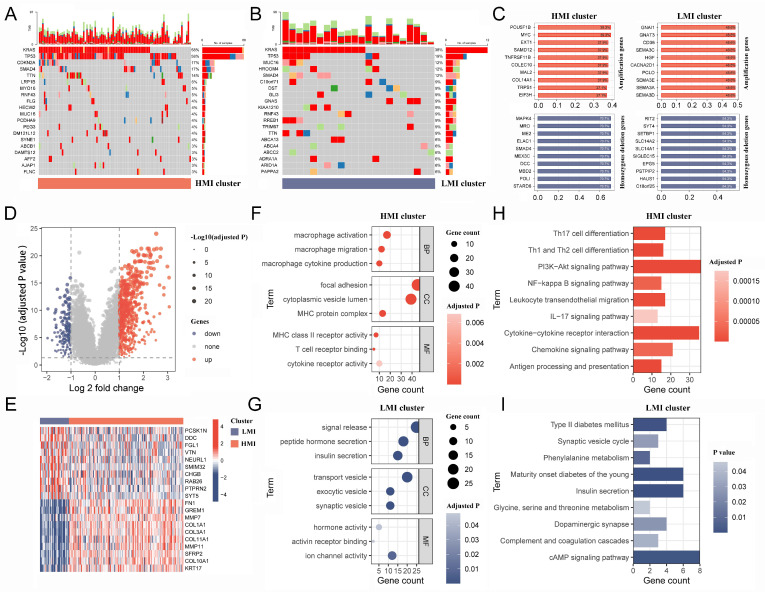
Mutation mapping and biological mechanisms in different clusters. (**A**,**B**). The waterfall plot of top 20 mutation genes in HMI and LMI clusters. (**C**). The top 10 amplification and HOMDEL genes in HMI and LMI clusters. (**D**). Volcano plot shows the DEGs between HMI and LMI clusters. (**E**) The heatmap shows top 10 up-regulated DEGs and top 10 down-regulated DEGs. (**F**,**G**). The bubble diagram shows the GO terms associated with the HMI and LMI clusters. (**H**,**I**). The bar chart shows the KEGG pathways involved in the HMI and LMI clusters. HMI, high-TAM2 infiltration; LMI, low-TAM2 infiltration; HOMDEL, homozygous deletion; DEGs, differentially expressed genes; GO, gene ontology; KEGG, Kyoto Encyclopedia of Genes and Genomes.

The pRRophetic R package was utilized to determine the IC50 values of commonly used chemotherapeutic agents for each sample. The results showed that PC patients with the HMI cluster were more sensitive to dasatinib, bortezomib, cyclophosphamide, vinblastine, and ruxolitinib (Figure 4F–J), while PC patients with the LMI cluster were more sensitive to entinostat, irinotecan oxaliplatin, vorinostat, and selumetinib (Figure 4K–O). Finally, we analyzed the somatic mutation data and CNV data of the clusters. By plotting the waterfall of the top 20 mutations in clusters, it was observed that within the top five mutations of high frequency, *TP53*, *KRAS*, *CDKN2A*, and *TTN* mutations were more frequent in the HMI cluster, while *MUC16* and *HROOM4* mutations were frequent in the LMI cluster (Figure 5A,B). Additionally, we investigated the amplifications and deletions of the copy numbers of the clusters, revealing that the homozygous deletions (HOMDEL) and amplifications were significantly different between the HMI and LMI clusters (Figure 5C).

### 2.6. Biological Mechanisms of Different Clusters

The results of the difference analysis indicated that 586 and 172 genes exhibited significant overexpression in the HMI and LMI clusters, respectively (Figure 5D, Supplementary Table S5), representing the top 10 characterized gene expression levels in both clusters in a heat map (Figure 5E). The biological mechanism of the HMI cluster exhibits a close association with macrophages and organismal immunity in the context of gene ontology (GO) enrichment analysis (Figure 5F, Supplementary Table S6), while the biological mechanism of the LMI cluster is related to pancreatic function and vesicular transport (Figure 5G, Supplementary Table S7). Based on the Kyoto Encyclopedia of Genes and Genomes (KEGG) pathway analysis results, the characteristic genes of the HMI cluster were found to participate in various classical tumor signaling pathways and immunological processes, including PI3K-Akt, NF-kappa B, and IL-17 signaling pathways (Figure 5H, Supplementary Table S8). However, the KEGG pathway involved in the LMI cluster was not associated with tumor development or immune response (Figure 5I, Supplementary Table S9).

### 2.7. Construction of a Prognostic Model Based on the TAM2-Driven Genes

**Figure 6:**
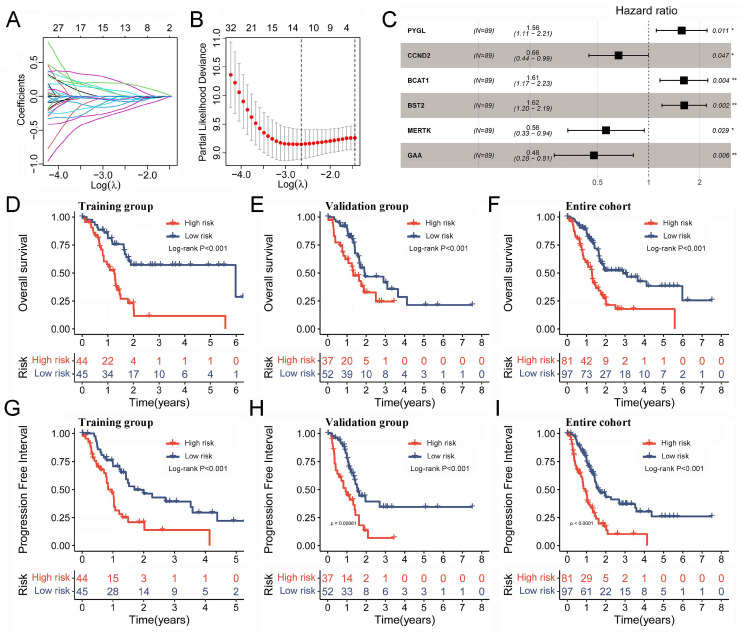
Prognostic model based on the TAM2-driven genes. (**A**) Distribution of Lasso coefficients of the prognostic TAM2-driven genes in the training group. (**B**) The cross-validation curve of Lasso regression shows the best penalty parameter value in the training group. (**C**) The 6 TAM2-driven genes construct the Cox risk model in the training group. (**D**–**F**) The OS time KM curve in the training group, validation group, and entire cohort. (**G**–**I**) The PFI time KM curve in the training group, validation group, and entire cohort. TAM2, tumor-associated macrophages M2; OS, overall survival; KM, Kaplan–Meier; PFI, progression-free interval. *, *p* < 0.05; **, *p* < 0.01.

**Figure 7:**
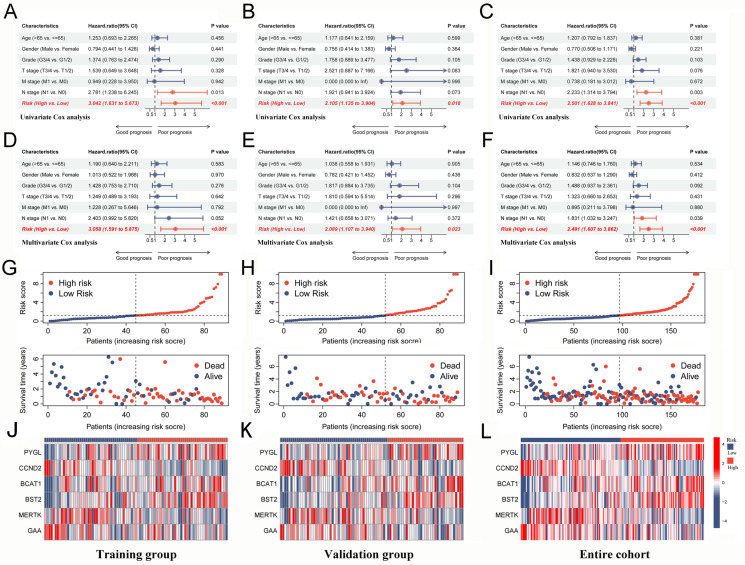
Risk score from Cox risk model is an independent predictor of prognosis in PC patients. (**A**–**C**) Univariate Cox analysis of risk score and clinical characteristics in the training group, validation group, and entire cohort. (**D**–**F**) Multivariate Cox analysis of risk score and clinical characteristics in the training group, validation group, and entire cohort. (**G**–**I**) The distribution trend of risk score, OS time in the training group, validation group and, entire cohort. (**J**–**L**) Expression levels of 6-gene signature in the high-risk and low-risk groups in the training group, validation group, and entire cohort. PC, pancreatic cancer; OS, overall survival.

The large number of TAM2-driven genes is not conducive to clinical detection. To address this issue, we developed a risk model utilizing Lasso–Cox regression analysis, which is based on TAM2-driven genes. Lasso regression analysis of the training groups showed that the optimal penalty parameter corresponded to TAM2-driven genes (Figure 6A,B). Therefore, the training groups were subjected to multivariate Cox regression analysis using these 13 genes. Finally, a Cox risk model consisting of six TAM2-driven genes was identified, calculating the risk score as follows: 0.447*PYGL − 0.409*CCND2 + 0.478*BCAT1 + 0.482*BST2 − 0.579*MERTK − 0.744*GAA (Figure 6C). Among the training, validation, and the entire groups, Kaplan–Meier survival curves revealed that the high-risk group exhibited shorter intervals of OS (Figure 6D–F) and PFI (Figure 6G–I). The study findings indicate that the risk score was identified as an independent prognostic factor for the patient outcomes in three datasets, as demonstrated by the univariate and multivariate Cox regression analyses (Figure 7A–F).

### 2.8. Assessing the Performance of a Prognostic Model

**Figure 8:**
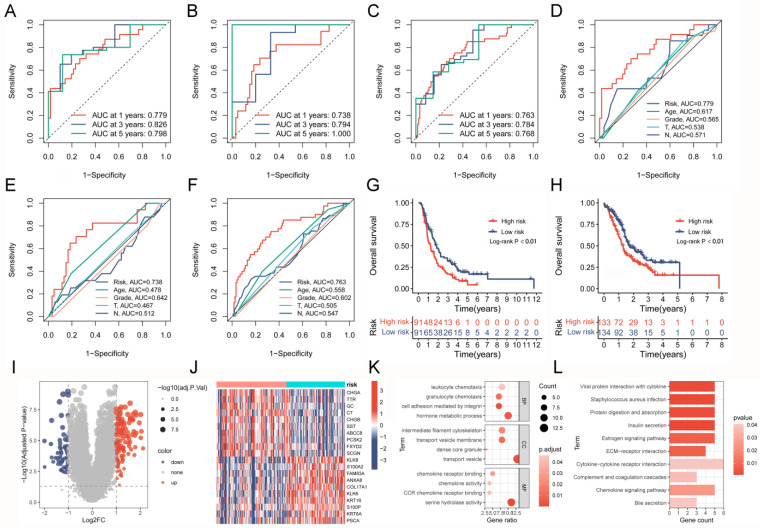
Assessing the prognostic predictive power and biological mechanisms of the Cox risk model. (**A**–**C**) ROC curves based on the risk model predict 1-, 3-, and 5-year OS rates in the training group, validation group, and entire cohort. (**D**–**F**) ROC curve of the risk model and clinical characteristics in the training group, validation group, and entire cohort. (**G**,**H**) Comparison of survival curves between high- and low-risk groups after applying the risk model to external datasets (ICGC-PACA-AU-array and ICGC-PACA-CA-seq). (**I**,**J**) Volcano plot and heatmap present the DEGs between high- and low-risk groups. (**K**,**L**) GO analysis and KEGG analysis results of risk score-related DEGs. ROC, Receiver Operating Characteristic; OS, overall survival; DEGs, differentially expressed genes; GO, gene ontology; KEGG, Kyoto Encyclopedia of Genes and Genomes.

The analysis of distribution patterns of risk scores and outcome status revealed a positive correlation between the risk scores of patients and the incidence of outcome events (Figure 7G–I). The expression profiles of six genes driven by TAM2 indicated that *PYGL*, *BCAT1*, and *BST2* manifested elevated expression levels in the high-risk group, whereas *CCND2*, *MERTK*, and *GAA* exhibited higher expression levels in the low-risk group (Figure 7J–L). The Cox risk model’s predictive ability was assessed using the Receiver Operating Characteristic (ROC) curve. The area under the curve (AUC) of risk score in the training group (0.779) was more elevated than other clinical features (Figure 8A), the validation group (AUC = 0.738) (Figure 8B), and the entire cohort (AUC = 0.763) (Figure 8C) to obtain consistent results. Moreover, a time-dependent survival ROC curve of the risk score was constructed for predicting 1-, 3-, and 5-year OS rates (Figure 8D–F). In order to enhance the credibility of the Cox risk model’s predictive ability, we conducted risk score calculations in two additional datasets (ICGC-PACA-AU-array and ICGC-PACA-CA-seq), both of which showed a worse prognosis for the high-risk group (Figure 8G,H). Differential analysis was conducted on the high- and low-risk groups to explore the potential mechanisms contributing to the unfavorable prognosis of patients in the high-risk group. The volcano plot illustrates that the high-risk group exhibited the up-regulation of 111 genes and down-regulation of 59 genes (Figure 8I, Supplementary Table S10). The heatmap shows the expression of each of the top 10 overexpressed and suppressed genes (Figure 8J). The enrichment analysis results suggested that differentially expressed genes are involved in the chemotactic movement of immune cells and immune molecules (Figure 8K,L, Supplementary Tables S11 and S12).

### 2.9. Confirmation of Critical TAM2-Driven Genes

**Figure 9:**
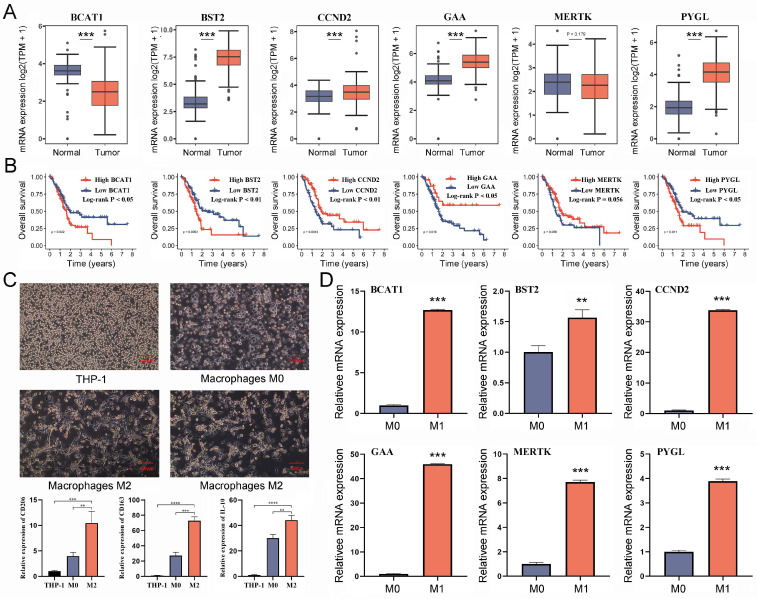
Validating the potential value of six TAM2-driven genes used to construct risk models in PC. (**A**) The differential expression of 6 genes in PC and normal pancreatic tissue was analyzed by the combination of TCGA-PAAD and GTEx-pancreatic cohorts. (**B**) KM survival curves demonstrate the prognostic significance of 6 genes in PC. (**C**) The expression levels of M2 macrophages markers were detected after induction of differentiation of THP-1 cells into M2 macrophages. (**D**) Comparison of the expression levels of 6 genes in M0 macrophages and M2 macrophages. TAM2, tumor-associated macrophages M2; PC, pancreatic cancer; KM, Kaplan–Meier. *, *p* < 0.05; **, *p* < 0.01; ***, *p* < 0.001, ****, *p* < 0.0001.

Given the good predictive performance of the Cox risk model, we further analyzed the characteristics of six model genes. The findings of the combined pancreatic cohorts of TCGA-PAAD and GTEx showed that *BST2*, *CCND2*, *GAA*, and *PYGL* were significantly overexpressed in PC tissues compared to normal pancreatic tissues, while the opposite was true for *BCAT1* (Figure 9A). The survival analysis results revealed that *BST2*, *CCND2*, *GAA*, *PYGL*, and *BCAT1* were all correlated to the prognosis of PC patients (Figure 9B). To determine whether the model genes are equally critical in M2 macrophages, we assayed the expression levels of the model gene in M2 macrophages. The THP-1 cells went through differentiation into M0 macrophages, which were subsequently differentiated into M2 macrophages (Figure 9C). The successful induction was confirmed by detecting the cellular markers *CD206/163* and *IL-10* of M2 macrophages (Figure 9C). Six model genes were shown to be significantly up-regulated in M2 macrophages when their expression levels were compared to those of M0 macrophages (Figure 9D). The results suggested that six model genes are the critical TAM2-driven genes involved in tumor progression.

## 3. Discussion

Pancreatic cancer is an intricate ailment resulting from the intricate interplay of various factors. Prior investigations have demonstrated that the genesis and progression of pancreatic cancer are concomitant with a plethora of genetic mutations, with key driver genes being *KRAS*, *CDKN2A*, *TP53*, and *SMAD4* [14]. Among these, the *KRAS* mutation stands as a pivotal initiating factor, signifying the metamorphosis from normal cells to initiated cells [15]. Additionally, *KRAS* mutation ranks among the most prevalent oncogenic events, occurring in approximately 90% of pancreatic cancer patients [16]. Nevertheless, genetic mutations or variations do not exclusively account for the etiology of pancreatic cancer; aberrant epigenetic modifications also foster the occurrence and development of the disease. Copious evidence points to the involvement of epigenetic modifications, such as DNA methylation, histone modifications, and RNA alterations, in the genesis and progression of pancreatic cancer [17]. Furthermore, afflictions affecting the pancreas itself, such as diabetes and chronic pancreatitis, may hasten the advancement of pancreatic cancer [18]. In recent years, with the advancement of high-throughput detection methods such as gene sequencing and multi-omics research, precision medicine has made significant strides in the diagnosis and treatment of pancreatic cancer. Precision medicine has greatly enhanced the efficacy of novel adjuvant and adjunctive therapies for pancreatic cancer through various aspects, including the exploration of critical therapeutic targets, the selection of appropriate patient populations, the assessment of treatment drug sensitivity and adverse reactions, and the monitoring of treatment dynamics, thereby extending patients’ survival time [19].

Tumor cells recruit peripheral blood mononuclear cells into tumor tissue and induce their differentiation into TAMs by secreting chemokines and cytokines. TAMs, widely considered as M2 subtypes, lead to immune evasion through producing inhibitory cytokines (IL-10 and TGF-β), the depletion of L-arginine metabolic activity, ROS production, and involvement of immune checkpoints [20,21]. TAMs enriched in the PC stroma could be potential targets for immunotherapy. Recent studies have shown that IFN-g enhances the efficacy of PD1-blocking therapies by inhibiting the infiltration of TAM2 [22]. Therefore, understanding the regulation of TAMs in the immunosuppressive microenvironment will help explore TAMs as adjuvant therapeutic targets for tumor immunotherapy. In the study, based on single-cell sequencing data and the WGCNA algorithm, TAM2-driven genes were identified that were positively correlated with TAM2 infiltration levels and characteristically up-regulated in TAM2. Two molecular subtypes of PC (HMI and LMI clusters) were obtained using unsupervised clustering based on TAM2-driven genes. TAMs are considered a prognostic indicator for many solid tumors, including PC, gastric, ovarian, and non-small-cell lung cancers [23,24,25]. A meta-analysis including 1699 PC patients showed that elevated levels of TAM2 infiltration predicted worse OS and disease-free survival [12]. In the same line with our results, PC patients in the HMI cluster had elevated levels of TAM2 infiltration and a poor prognosis. In addition, PC patients in the HMI cluster have features of advanced tumors, including poor histological grade and N stage. The lower level of tumor cell differentiation in the HMI cluster may be related to TAM2 promoting the stemness of tumor cells [26]. Cytokines and chemokines derived from TAM2, such as IL1/8 and CCL18, can enhance the epithelial–mesenchymal transition in tumor tissues through various signaling pathways, which leads to advanced tumor invasion and metastasis [27,28].

Many studies have identified that TAM2 plays a crucial role in the PC microenvironment by driving the immunosuppressive cell to polarize and expand, and we also found abnormally elevated levels of Tregs infiltration in the HMI cluster. TAM2 induces CD4 T cell differentiation into tumor-promoting Tregs while promoting Treg aggregation in the tumor to suppress the antitumor response [29]. In our study, the level of Tregs infiltration exhibited a significant elevation in the HMI cluster compared to the LMI cluster. Given the regulatory role that TAM2 plays in the immunosuppressive microenvironment of PC, we further explored the response to immunotherapy in different clusters. As expected, the HMI cluster exhibited strong resistance and a low response rate to ICI treatment due to high levels of TAM2 infiltration. This result suggests that the application of ICI therapy, along with the inhibition of TAM2 polarization and recruitment, may provide a better prognosis for PC patients. In addition, to characterize the molecular profile of clusters, we investigated genomic alterations. The HMI cluster has a higher frequency of mutations in two tumor-associated genes, *KRAS* and *TP53*, which partly explains the poor prognosis and malignant progression of patients in the HMI cluster.

The emergence of multi-omics data has led to the discovery of numerous genetic traits and risk models, which offer innovative perspectives on tumor diagnosis and prognosis prediction [30,31]. Multiple genetic features are necessary for accurate and reliable predictive models in tumor biology due to their complexity. Relying on a single pathological feature or biomarker is insufficient. We constructed a Cox risk model that could predict the prognosis of PC patients and identify the hub genes in TAM2-driven genes. The Cox risk model consists of six TAM2-driven genes that are employed for the calculation of risk score for each patient suffering from PC, which is consequently used to categorize the patients into high- and low-risk groups. The survival analysis results suggested a significant difference in prognosis between the two risk groups. Additionally, the AUC demonstrated the predictive power of the Cox risk model. The validation group and the whole group were both used to validate the model. Furthermore, the Cox risk model went through external validation through the ICGC-PACA-AU-array and ICGC-PACA-CA-seq cohorts, which produced comparable predictive capabilities.

We identified six hub TAM2-driven genes by the Cox risk model: *PYGL*, *CCND2*, *BCAT1*, *BST2*, *MERTK*, and *GAA*. *PYGL* is a key gene involved in the glycolytic pathway with glycogen phosphorylase activity. A preclinical study showed that *PYGL* induces EMT and metastasis in PC by stimulating the glycolysis of glycogen accumulated in tumor cells [32]. Cyclin D2 is encoded by *CCND2* and plays a role in cell cycle progression, and is believed to regulate cyclin-dependent kinases 4/6 during the G1-S transition [33]. *CCND2* is dysregulated in various tumors and is correlated to patient prognosis, including lung, breast, and liver cancers [34]. Silva et al. reported that the catabolism of branched-chain amino acids plays an immunomodulatory function in glioblastoma, which is associated with the inhibition of macrophage phagocytic activity [35]. *BCAT1* plays an important role as a transaminase in the catabolism of branched-chain amino acids. *BCAT1* is the most abundant isoform in human macrophages and is involved in regulating the activation of pro-inflammatory macrophages [36]. *BST2*, a type II transmembrane protein, exhibits oncogenic properties in diverse tumors such as PC, myeloma, breast, lung, and kidney cancers [37,38,39]. A preclinical study in colorectal cancer showed that up-regulated *BST2* promotes immunosuppressive TME by recruiting TAMs and inducing them into the M2 phenotype [40]. In addition, *FGD5-AS1*-regulated *BST2* plays a role in promoting M2 macrophage polarization and inhibiting M1 macrophage polarization in cervical cancer [41]. *MERTK* is a novel type I receptor tyrosine kinase that is widely expressed in macrophages and promotes phagocytosis [42]. After recognizing ligands exposed on the surface of apoptotic cells, *MERTK* rapidly mediates the phagocytosis and macrophage clearance of apoptotic cells, a process known as efferocytosis [43]. During tumor development, *MERTK* promotes immune evasion and the M2 polarization of macrophages through the regulation of efferocytosis [44]. *GAA* is involved in regulating the autophagic process in tumor cells. A recent study showed that low levels of *GAA* induced autophagy in gastric cancer cells through AMPK activation and inhibition of mTORC1 signaling. In contrast, high levels of *GAA* were observed to inhibit autophagy by reducing intracellular ATP levels and the number of active lysosomes [45].

## 4. Materials and Methods

### 4.1. Data Acquisition

The R 4.0.5 software was utilized for the purposes of data processing, statistical analysis, and visualization. The study objectives were met by collecting datasets from various databases, including the Cancer Genome Atlas (TCGA) and the International Cancer Genome Consortium (ICGC), the Genotype-Tissue Expression (GTEx) and Gene Expression Omnibus (GEO) databases [46,47,48,49]. Transcriptome data and clinical information of PC samples include three study cohorts: TCGA-PAAD (n = 178), ICGC-PACA-AU-array (n = 167), and ICGC-PACA-CA-seq (n = 182). Transcriptome data of normal pancreatic tissue were from the GTEx-pancreatic cohort (n = 167). Single-cell sequencing data of PC samples were from GSE154778 (n = 16). Somatic mutation files for the TCGA-PAAD cohort were obtained by accessing the UCSC database, and the maftools R package was used for reading, organizing, and visualizing. The copy number variation (CNV) data from the TCGA-PAAD cohort were obtained by accessing the cBioportal database [50,51].

### 4.2. Immune Cell Infiltration Analysis

Analysis of the TCGA-PAAD cohort using the CIBERSORT R package enabled the capture of 22 TIC levels in each PC sample [52]. TICs that exhibited an infiltration level of 0 in 50% of the PC samples were removed, and 14 TICs were finally retained for further analysis.

### 4.3. Weighted Correlation Network Analysis (WGCNA)

The study utilized the WGCNA R package for building a weighted co-expression network and filtering co-expression modules [53]. The samples with a large dispersion were removed by hierarchical clustering. The soft power of k = 12 was selected. Subsequently, the transformation of the expression matrix into a topology matrix was executed. The hybrid dynamic shearing tree standard was employed to cluster genes using the average-linkage hierarchical clustering approach, according to TOM. The modules that were in close proximity were combined into novel modules. The correlation between each module and TIC infiltration of the samples was calculated. Finally, the module genes with the highest module–TAM2 associations were identified as the TAM2 co-expression genes.

### 4.4. Single-Cell Sequencing Data Download and Processing

First, ineligible cells and genes were removed following these criteria: (1) cells expressing <200 genes; (2) genes expressed in <3 cells; (3) cells having a percentage of mitochondrial or erythrocyte gene percentage >5%; (4) cells having a number of expressed genes >7500. Seurat R package and single R package were used for the full-flow analysis and cell annotation of single-cell sequencing data [54]. Finally, the FindMarkers function was used to complete the differential analysis between TAMs and other cells. The criteria used to screen significantly differentially expressed genes (DEGs) were as follows: Log2 fold change (Log2FC) > 0.1 and adjusted *p* value < 0.05.

### 4.5. Consensus Clustering

Extraction of the overlapping regions of TAM2 co-expression genes and DEGs as TAM2 driver genes was carried out. Based on the TAM2-driven genes, consensus clustering was further carried out in the TCGA-PAAD cohort using the ConsensusClusterPlus R package [55]. The study set the parameters as the Euclidean distance-based Pam algorithm with 1000 iterations. At each iteration, 80% of the samples went through drawing in a random manner. The selection of an optimal number of clusters relied on CDF and ranged from 2 to 9 clusters. Finally, 2 clusters were identified, named cluster high-TAM2 infiltration (HMI) and cluster low-TAM2 infiltration (LMI).

### 4.6. Targeted Drug Sensitivity Analysis and Immunotherapy Prediction

The medication sensitivity of each patient with PC was estimated according to their gene expression profiles using the Oncopredict R package [56,57]. The study utilized the Wilcoxon Test functions in R for screening potential drug sensitivity within HMI and LMI clusters, whereby lower IC50 values were indicative of increased drug sensitivity. This study also accessed the TIDE and TCIA databases not only to assess the potential for resistance to immunotherapy and the response to ICI treatment for the HMI and LMI clusters [58,59], but also to obtain the TIDE and immunophenoscore (IPS) scores of PC patients, respectively. A positive correlation was observed between elevated IPS and reduced TIDE scores, indicating a favorable immunotherapeutic outcome.

### 4.7. Functional Enrichment Analysis of Different Clusters

We indirectly analyzed the biological functions involved in HMI and LMI clusters using the molecular characterization of clusters. First, DEGs of different clusters were identified through the Limma R package. The molecular characteristics of HMI clusters were defined as Log2FC > 1 and adj-*p* < 0.05. The molecular characteristics of LMI clusters were defined as Log2FC < 1 and adj-*p* < 0.05. The enrichKEGG and enrichGO functions in the clusterProfiler R package were then used to analyze the KEGG pathways and GO terms involved in the molecular characteristics of different clusters [60].

### 4.8. COX Risk Model Construction

The TCGA-PAAD cohort was randomly assigned to a training group (n = 89) and a validation group (n = 89) using the caret R package. The COX risk model, which was based on TAM2-driven genes, was constructed using the data from the training group. The TAM2-driven genes were analyzed using univariate COX regression analysis according to OS, and those genes that exhibited significant prognostic significance were identified by screening (*p* < 0.05). To mitigate the risk of overfitting, the study used the least absolute shrinkage and selection operator (LASSO) regression to evaluate potential prognostic genes. The list of prognostic genes corresponding to the optimal penalty parameter is obtained when the cross-validation error of Lasso regression is the smallest. The LASSO prognostic genes went through a multivariate COX regression analysis to determine the ultimate risk model. The risk score for each patient was calculated as follows: risk score = βmRNA1 × ExpressionmRNA1 + βmRNA2 × ExpressionmRNA2 + …+βmRNAn × ExpressionmRNAn.

### 4.9. Cell Culture and Treatments

The THP-1 cells (Shanghai Institute of Nutrition and Health, Shanghai, China) were cultured in Dulbecco’s modified Eagle medium (DMEM, Gibco, Grand Island, NY, USA) with 10% fetal bovine serum (FBS, Gibco) and incubated at 37 °C under 5% CO_2_. The THP-1 monocytes went through a 72 h treatment of 100 ng/mL PMA (Abcam, Cambridge, MA, USA), resulting in their conversion into M0 macrophages. Subsequently, these macrophages were subjected to a 48 h incubation with 20 ng/mL IL-4 (Peprotech, Rocky Hill, NJ, USA), leading to the acquisition of M2 macrophages.

### 4.10. RNA Extraction and qRT-PCR

The RNA was extracted from the samples utilizing TRIzol, followed by the utilization of PrimeScript RT Reagent Kit to synthesize cDNA. qRT-PCR analyses were performed using a StepOne real-time PCR instrument (Applied Biosystems, Nyack, NY, USA). The 2^−ΔΔCt^ method was used to analyze relative gene expression, with the purpose of normalizing the data, which was achieved by the use of GAPDH. Supplementary Table S13 lists the used primer sequences. All analyses were repeated three times.

### 4.11. Statistical Analysis

Statistical calculations and the visualization of all results were conducted through R (version 4.0.2, R Foundation, Vienna, Austria). The statistical analysis of categorical variables involved the utilization of either the chi-square test or Fisher’s exact test. The statistical differences in the measured variables were analyzed using the Wilcoxon rank-sum test. Spearman’s rank correlation coefficient was employed to assess the association between the two variables. The prognostic analysis was conducted utilizing the Kaplan–Meier survival curve, as well as univariate and multivariate Cox analyses. *p* < 0.05 indicated a significant difference.

## 5. Conclusions

In conclusion, we classified PC depending on TAM2-driven gene expressions. The immune subtypes exhibit significant differences in TIC infiltration, prognosis, and immunotherapy effects. A six-gene prognostic model was constructed relying on these TAM2-driven gene expressions. The 6-gene signature exhibits robust stability and demonstrates consistent predictive efficacy across diverse databases. Finally, we identified that BCAT1, BST2, and MERTK expression levels were significantly increased in TAM2 as potential mechanistic molecules that induce the polarization of TAMs toward the M2 phenotype.

## Data Availability

The datasets presented in this study (sourced from TCGA, ICGC and GEO) can be found in online repositories. The datasets sourced from clinical samples are available from the corresponding author on reasonable request.

## Supplementary Materials

The following supporting information can be downloaded at: https://www.mdpi.com/article/10.3390/ijms241612787/s1.

## References

[1] Grossberg A.J., Chu L.C., Deig C.R., Fishman E.K., Hwang W.L., Maitra A., Marks D.L., Mehta A., Nabavizadeh N., Simeone D.M. (2020). Multidisciplinary standards of care and recent progress in pancreatic ductal adenocarcinoma. CA Cancer J. Clin..

[2] Siegel R.L., Miller K.D., Fuchs H.E., Jemal A. (2022). Cancer statistics, 2022. CA Cancer J. Clin..

[3] Hidalgo M., Cascinu S., Kleeff J., Labianca R., Löhr J.M., Neoptolemos J., Real F.X., Van Laethem J.L., Heinemann V. (2015). Addressing the challenges of pancreatic cancer: Future directions for improving outcomes. Pancreatology.

[4] Barugola G., Partelli S., Marcucci S., Sartori N., Capelli P., Bassi C., Pederzoli P., Falconi M. (2009). Resectable pancreatic cancer: Who really benefits from resection?. Ann. Surg. Oncol..

[5] Yang J., Li Y., Sun Z., Zhan H. (2021). Macrophages in pancreatic cancer: An immunometabolic perspective. Cancer Lett..

[6] Wilson R., Kinloch E., Makaroff L.E., Bailey-Bearfield A., Stephens R., Rawlinson J., Oliver K. (2023). A major conditions strategy cannot replace a national cancer plan-patient advocates voice their concerns. Lancet Oncol..

[7] Liu Q., Liao Q., Zhao Y. (2017). Chemotherapy and tumor microenvironment of pancreatic cancer. Cancer Cell Int..

[8] Feig C., Gopinathan A., Neesse A., Chan D.S., Cook N., Tuveson D.A. (2012). The pancreas cancer microenvironment. Clin. Cancer Res..

[9] Beatty G.L., Eghbali S., Kim R. (2017). Deploying Immunotherapy in Pancreatic Cancer: Defining Mechanisms of Response and Resistance. Am. Soc. Clin. Oncol. Educ. Book.

[10] Poh A.R., Ernst M. (2021). Tumor-Associated Macrophages in Pancreatic Ductal Adenocarcinoma: Therapeutic Opportunities and Clinical Challenges. Cancers.

[11] Loeuillard E., Yang J., Buckarma E., Wang J., Liu Y., Conboy C., Pavelko K.D., Li Y., O’Brien D., Wang C. (2020). Targeting tumor-associated macrophages and granulocytic myeloid-derived suppressor cells augments PD-1 blockade in cholangiocarcinoma. J. Clin. Investig..

[12] Yu M., Guan R., Hong W., Zhou Y., Lin Y., Jin H., Hou B., Jian Z. (2019). Prognostic value of tumor-associated macrophages in pancreatic cancer: A meta-analysis. Cancer Manag. Res..

[13] Locati M., Curtale G., Mantovani A. (2020). Diversity, Mechanisms, and Significance of Macrophage Plasticity. Annu. Rev. Pathol..

[14] Wood L.D., Canto M.I., Jaffee E.M., Simeone D.M. (2022). Pancreatic Cancer: Pathogenesis, Screening, Diagnosis, and Treatment. Gastroenterology.

[15] Storz P., Crawford H.C. (2020). Carcinogenesis of Pancreatic Ductal Adenocarcinoma. Gastroenterology.

[16] Jones S., Zhang X., Parsons D.W., Lin J.C., Leary R.J., Angenendt P., Mankoo P., Carter H., Kamiyama H., Jimeno A. (2008). Core signaling pathways in human pancreatic cancers revealed by global genomic analyses. Science.

[17] Gao J., Wang L., Xu J., Zheng J., Man X., Wu H., Jin J., Wang K., Xiao H., Li S. (2013). Aberrant DNA methyltransferase expression in pancreatic ductal adenocarcinoma development and progression. J. Exp. Clin. Cancer Res. CR.

[18] Menini S., Iacobini C., de Latouliere L., Manni I., Vitale M., Pilozzi E., Pesce C., Cappello P., Novelli F., Piaggio G. (2020). Diabetes promotes invasive pancreatic cancer by increasing systemic and tumour carbonyl stress in Kras(G12D/+) mice. J. Exp. Clin. Cancer Res. CR.

[19] Froeling F.E.M., Casolino R., Pea A., Biankin A.V., Chang D.K. (2021). Molecular Subtyping and Precision Medicine for Pancreatic Cancer. J. Clin. Med..

[20] Pan Y., Yu Y., Wang X., Zhang T. (2020). Tumor-Associated Macrophages in Tumor Immunity. Front. Immunol..

[21] Yan S., Wan G. (2021). Tumor-associated macrophages in immunotherapy. FEBS J..

[22] Zhang M., Huang L., Ding G., Huang H., Cao G., Sun X., Lou N., Wei Q., Shen T., Xu X. (2020). Interferon gamma inhibits CXCL8-CXCR2 axis mediated tumor-associated macrophages tumor trafficking and enhances anti-PD1 efficacy in pancreatic cancer. J. Immunother. Cancer.

[23] Liao Z., Ye L., Li T., Jin X., Lin X., Fei Q., Zhang H., Shi S., Yu X., Jin K. (2023). Tissue-resident CXCR4(+) macrophage as a poor prognosis signature promotes pancreatic ductal adenocarcinoma progression. Int. J. Cancer.

[24] Ohno S., Inagawa H., Dhar D.K., Fujii T., Ueda S., Tachibana M., Ohno Y., Suzuki N., Inoue M., Soma G. (2005). Role of tumor-associated macrophages (TAM) in advanced gastric carcinoma: The impact on FasL-mediated counterattack. Anticancer Res..

[25] Yuan X., Zhang J., Li D., Mao Y., Mo F., Du W., Ma X. (2017). Prognostic significance of tumor-associated macrophages in ovarian cancer: A meta-analysis. Gynecol. Oncol..

[26] Valle S., Martin-Hijano L., Alcalá S., Alonso-Nocelo M., Sainz B. (2018). The Ever-Evolving Concept of the Cancer Stem Cell in Pancreatic Cancer. Cancers.

[27] Chen S.J., Lian G.D., Li J.J., Zhang Q.B., Zeng L.J., Yang K.G., Huang C.M., Li Y.Q., Chen Y.T., Huang K.H. (2018). Tumor-driven like macrophages induced by conditioned media from pancreatic ductal adenocarcinoma promote tumor metastasis via secreting IL-8. Cancer Med..

[28] Tekin C., Aberson H.L., Waasdorp C., Hooijer G.K.J., de Boer O.J., Dijk F., Bijlsma M.F., Spek C.A. (2020). Macrophage-secreted MMP9 induces mesenchymal transition in pancreatic cancer cells via PAR1 activation. Cell. Oncol..

[29] Daley D., Mani V.R., Mohan N., Akkad N., Pandian G., Savadkar S., Lee K.B., Torres-Hernandez A., Aykut B., Diskin B. (2017). NLRP3 signaling drives macrophage-induced adaptive immune suppression in pancreatic carcinoma. J. Exp. Med..

[30] Huntley C., Torr B., Sud A., Rowlands C.F., Way R., Snape K., Hanson H., Swanton C., Broggio J., Lucassen A. (2023). Utility of polygenic risk scores in UK cancer screening: A modelling analysis. Lancet Oncol..

[31] Mela A., Rdzanek E., Tysarowski A., Sakowicz M., Jaroszyński J., Furtak-Niczyporuk M., Żurek G., Poniatowski Ł.A., Jagielska B. (2023). The impact of changing the funding model for genetic diagnostics and improved access to personalized medicine in oncology. Expert Rev. Pharm. Outcomes Res..

[32] Ji Q., Li H., Cai Z., Yuan X., Pu X., Huang Y., Fu S., Chu L., Jiang C., Xue J. (2023). PYGL-mediated glucose metabolism reprogramming promotes EMT phenotype and metastasis of pancreatic cancer. Int. J. Biol. Sci..

[33] Ortega S., Malumbres M., Barbacid M. (2002). Cyclin D-dependent kinases, INK4 inhibitors and cancer. Biochim. Biophys. Acta.

[34] Hung C.S., Wang S.C., Yen Y.T., Lee T.H., Wen W.C., Lin R.K. (2018). Hypermethylation of CCND2 in Lung and Breast Cancer Is a Potential Biomarker and Drug Target. Int. J. Mol. Sci..

[35] Silva L.S., Poschet G., Nonnenmacher Y., Becker H.M., Sapcariu S., Gaupel A.C., Schlotter M., Wu Y., Kneisel N., Seiffert M. (2017). Branched-chain ketoacids secreted by glioblastoma cells via MCT1 modulate macrophage phenotype. EMBO Rep..

[36] Ko J.H., Olona A., Papathanassiu A.E., Buang N., Park K.S., Costa A.S.H., Mauro C., Frezza C., Behmoaras J. (2020). BCAT1 affects mitochondrial metabolism independently of leucine transamination in activated human macrophages. J. Cell Sci..

[37] Lei C., Hou Y., Chen J. (2021). Specificity protein 1-activated bone marrow stromal cell antigen 2 accelerates pancreatic cancer cell proliferation and migration. Exp. Ther. Med..

[38] Laoui D., Van Overmeire E., Movahedi K., Van den Bossche J., Schouppe E., Mommer C., Nikolaou A., Morias Y., De Baetselier P., Van Ginderachter J.A. (2011). Mononuclear phagocyte heterogeneity in cancer: Different subsets and activation states reaching out at the tumor site. Immunobiology.

[39] Schouppe E., De Baetselier P., Van Ginderachter J.A., Sarukhan A. (2012). Instruction of myeloid cells by the tumor microenvironment: Open questions on the dynamics and plasticity of different tumor-associated myeloid cell populations. Oncoimmunology.

[40] He X., Chen H., Zhong X., Wang Y., Hu Z., Huang H., Zhao S., Wei P., Shi D., Li D. (2023). BST2 induced macrophage M2 polarization to promote the progression of colorectal cancer. Int. J. Biol. Sci..

[41] Liu G., Du X., Xiao L., Zeng Q., Liu Q. (2021). Activation of FGD5-AS1 Promotes Progression of Cervical Cancer through Regulating BST2 to Inhibit Macrophage M1 Polarization. J. Immunol. Res..

[42] Lahey K.C., Gadiyar V., Hill A., Desind S., Wang Z., Davra V., Patel R., Zaman A., Calianese D., Birge R.B. (2022). Mertk: An emerging target in cancer biology and immuno-oncology. Int. Rev. Cell Mol. Biol..

[43] Rothlin C.V., Carrera-Silva E.A., Bosurgi L., Ghosh S. (2015). TAM receptor signaling in immune homeostasis. Annu. Rev. Immunol..

[44] Stanford J.C., Young C., Hicks D., Owens P., Williams A., Vaught D.B., Morrison M.M., Lim J., Williams M., Brantley-Sieders D.M. (2014). Efferocytosis produces a prometastatic landscape during postpartum mammary gland involution. J. Clin. Investig..

[45] Liu Y., Ma Y., Li Z., Yang Y., Yu B., Zhang Z., Wang G. (2020). Investigation of Inhibition Effect of Gossypol-Acetic Acid on Gastric Cancer Cells Based on a Network Pharmacology Approach and Experimental Validation. Drug Des. Dev. Ther..

[46] Weinstein J.N., Collisson E.A., Mills G.B., Shaw K.R., Ozenberger B.A., Ellrott K., Shmulevich I., Sander C., Stuart J.M. (2013). The Cancer Genome Atlas Pan-Cancer analysis project. Nat. Genet..

[47] Lonsdale J., Thomas J., Salvatore M., Phillips R., Lo E., Shad S., Hasz R., Walters G., Garcia F., Young N. (2013). The Genotype-Tissue Expression (GTEx) project. Nat. Genet..

[48] Zhang J., Bajari R., Andric D., Gerthoffert F., Lepsa A., Nahal-Bose H., Stein L.D., Ferretti V. (2019). The International Cancer Genome Consortium Data Portal. Nat. Biotechnol..

[49] Barrett T., Wilhite S.E., Ledoux P., Evangelista C., Kim I.F., Tomashevsky M., Marshall K.A., Phillippy K.H., Sherman P.M., Holko M. (2013). NCBI GEO: Archive for functional genomics data sets—update. Nucleic Acids Res..

[50] Mayakonda A., Lin D.C., Assenov Y., Plass C., Koeffler H.P. (2018). Maftools: Efficient and comprehensive analysis of somatic variants in cancer. Genome Res..

[51] Gao J., Aksoy B.A., Dogrusoz U., Dresdner G., Gross B., Sumer S.O., Sun Y., Jacobsen A., Sinha R., Larsson E. (2013). Integrative analysis of complex cancer genomics and clinical profiles using the cBioPortal. Sci. Signal.

[52] Kim Y., Kang J.W., Kang J., Kwon E.J., Ha M., Kim Y.K., Lee H., Rhee J.K., Kim Y.H. (2021). Novel deep learning-based survival prediction for oral cancer by analyzing tumor-infiltrating lymphocyte profiles through CIBERSORT. Oncoimmunology.

[53] Langfelder P., Horvath S. (2008). WGCNA: An R package for weighted correlation network analysis. BMC Bioinform..

[54] Mangiola S., Doyle M.A., Papenfuss A.T. (2021). Interfacing Seurat with the R tidy universe. Bioinformatics.

[55] Wilkerson M.D., Hayes D.N. (2010). ConsensusClusterPlus: A class discovery tool with confidence assessments and item tracking. Bioinformatics.

[56] Maeser D., Gruener R.F., Huang R.S. (2021). oncoPredict: An R package for predicting in vivo or cancer patient drug response and biomarkers from cell line screening data. Brief. Bioinform..

[57] Mela A., Poniatowski Ł.A., Drop B., Furtak-Niczyporuk M., Jaroszyński J., Wrona W., Staniszewska A., Dąbrowski J., Czajka A., Jagielska B. (2020). Overview and Analysis of the Cost of Drug Programs in Poland: Public Payer Expenditures and Coverage of Cancer and Non-Neoplastic Diseases Related Drug Therapies from 2015–2018 Years. Front. Pharmacol..

[58] Jiang P., Gu S., Pan D., Fu J., Sahu A., Hu X., Li Z., Traugh N., Bu X., Li B. (2018). Signatures of T cell dysfunction and exclusion predict cancer immunotherapy response. Nat. Med..

[59] Charoentong P., Finotello F., Angelova M., Mayer C., Efremova M., Rieder D., Hackl H., Trajanoski Z. (2017). Pan-cancer Immunogenomic Analyses Reveal Genotype-Immunophenotype Relationships and Predictors of Response to Checkpoint Blockade. Cell Rep..

[60] Yu G., Wang L.G., Han Y., He Q.Y. (2012). clusterProfiler: An R package for comparing biological themes among gene clusters. Omics.

